# Variability of efavirenz plasma concentrations among pediatric HIV patients treated with efavirenz based combination antiretroviral therapy in Dar es Salaam, Tanzania

**DOI:** 10.1186/s40360-018-0258-6

**Published:** 2018-10-23

**Authors:** Selemani Saidi Sungi, Eliford Ngaimisi, Nzovu Ulenga, Philip Sasi, Sabina Mugusi

**Affiliations:** 1 Health Department, Chamwino District Council, Chamwino, Dodoma, Tanzania; 2 Unit of Pharmacology and Therapeutics, School of Pharmacy, Muhimbili University of Health and Allied Sciences (MUHAS), Dar es Salaam, Tanzania; 3 Management Development for Health (MDH), Dar es Salaam, Tanzania; 4 Department of Clinical Pharmacology, School of Medicine, Muhimbili University of Health and Allied Sciences (MUHAS), Dar es Salaam, Tanzania

**Keywords:** cART, Efavirenz, Variability, Tanzania

## Abstract

**Background:**

Children are subject to varying drug pharmacokinetics which influence plasma drug levels, and hence treatment outcomes especially for drugs like efavirenz whose plasma concentrations are directly related to treatment outcomes. This study is aimed at determining plasma efavirenz concentrations among Tanzanian pediatric HIV-1 patients on efavirenz-based combination antiretroviral therapy (cART) and relating it to clinical, immunological and virologic treatment responses.

**Methods:**

A cross sectional study involving pediatric HIV patients aged 5–15 years on efavirenz-based cART for ≥ 6 months were recruited in Dar es Salaam. Data on demographics, cART regimens, efavirenz dose and time of the last dose were collected using structured questionnaires and checklists. Venous blood samples were drawn at 10–19 h post-dosing for efavirenz plasma analysis.

**Results:**

A total of 145 children with a mean ± SD age of 10.83 ± 2.75 years, on cART for a mean ± SD of 3.7 ± 2.56 years were recruited. Median [IQR] efavirenz concentration was 2.56 [IQR = 1.5–4.6] μg/mL with wide inter-patient variability (CV 111%). Poor virologic response was observed in 70.8%, 20.8% and 15.9% of patients with efavirenz levels < 1 μg/mL, 1–4 μg/mL and > 4 μg/mL respectively. Patients with efavirenz levels of < 1 μg/mL were 11 times more likely to have detectable viral loads. Immunologically, 31.8% of children who had low levels (< 1 μg/mL) of efavirenz had a CD4 count of < 350 cells/μL.

**Conclusion:**

Wide inter-individual variability in efavirenz plasma concentrations is seen among Tanzanian children in routine clinical practice with many being outside the recommended therapeutic range. Virologic failure is very high in children with sub-therapeutic levels. Concentrations outside the therapeutic window suggest the need for dose adjustment on the basis of therapeutic drug monitoring to optimize treatment.

## Background

Combination antiretroviral therapy (cART) has revolutionized the lives of HIV-1infected adults and children across the world contributing to the continual decrease of new infections [1]. With adequate resources, management of pediatric HIV infection using cART has shown substantial clinical benefits and improved quality of life such as improvement in immunologic status, sustained virologic suppression and enhancement of survival. Such favorable responses are similar to those observed in adults, however, these benefits are observed when optimal plasma drug concentrations of cART drugs are attained and maintained [2].

Treatment for HIV-1 infections in Tanzania involves the use of a combination of antiretroviral drugs commonly with two Nucleoside Reverse Transcriptase Inhibitors (NRTI) and one Non-Nucleoside Reverse Transcriptase Inhibitor (NNRTI) [3]. The National AIDS Control Program (NACP) guidelines currently recommend protease inhibitor (PI)-based regimens for all pediatric HIV patients previously exposed to nevirapine (an NNRTI) during prevention of mother to child transmission. The recommended regimen for the under-three-years-old pediatric patients now is abacavir, lamivudine and lopinavir/ritonavir (a protease inhibitor - based cART) [3]. After reaching three years of age the protease inhibitor is replaced by an NNRTI particularly efavirenz.

The use of efavirenz-based regimens among pediatric patients aged at least three years and above is an advantage in resource-constrained settings because efavirenz has fewer drug interactions compared to protease inhibitors and appears to be better tolerated with less risk of leading to severe adverse effects than nevirapine [4–7]. This has led to better treatment outcomes compared to nevirapine making it a better NNRTI option, with recommendations from the world health organization (WHO) to make efavirenz the NNRTI of choice in first line treatment of HIV-1 infections [4, 5, 8]. More recent research has shown the safety of prolonged cART use among HIV-infected children and suggest that suppressive NNRTI-based regimens can be associated with lower levels of systemic inflammation [9]. Efavirenz is a key drug in the treatment of HIV infection among pediatric patients aged three years and above in Tanzania [3].

Efavirenz is available in both liquid and solid formulations (suspension and tablet/capsule). For patients older than three years efavirenz dosing is in accordance with weight bands starting with children weighing at least 10Kg [3]. Both inter and intra-individual variability in pharmacokinetics of efavirenz leads to variability in efavirenz steady state plasma concentrations. Concentrations above 4 μg/mL are normally associated with increased central nervous system (CNS) adverse effects such as insomnia, frequent nightmares and hallucinations, whereas efavirenz plasma concentrations below 1 μg/mL result into more frequent treatment failures [10] . Some of the factors associated with efavirenz pharmacokinetic variability include; host genetic factors, body weight, gender, ethnicity, drug interactions and binding to plasma proteins [8, 11–13]. The appropriate use of cART in pediatric patients requires careful considerations of individual drugs’ disposition kinetics, as well as the impact on the drugs’ pharmacokinetics and pharmacodynamics occurring during developmental changes as a child grows [14].

These variations in pharmacokinetic parameters lead to unpredictable responses to treatment in pediatric patients [15]. Therefore, even with the use of pediatric fixed-dose combination antiretroviral tablets, treatment outcome may still be suboptimal in a considerable proportion of patients [16]. This variability may lead to sub-therapeutic or supra-therapeutic concentrations of efavirenz in pediatric patients which is a major threat to the long-term success of antiretroviral treatment. Resulting sub-therapeutic concentrations may be associated with lack of potency in suppressing viral replication leading to an increased chance of developing mutations, subsequently resistance, and hence treatment failure [13]. Supra-therapeutic concentrations increase the risk for toxicity, poor adherence and eventual treatment failure as well.

Treatment failures among pediatric patients on efavirenz-based cART are still observed in our settings as evidenced by pediatric patients being switched to alternative first line regimens or to the more complex and costly second-line regimens. This may be associated with children not achieving therapeutic efavirenz concentrations which leads to inadequate suppression of viral replication and hence treatment failure. Resistance and eventual treatment failure is of great concern because of fewer first line alternative options in our settings as well as the high cost of second line regimens (which are complex and may not be available in our settings) [17]. This is particularly important for children who will be in need of cART for their whole lives.

## Methods

### Study sites and study design

This was a cross sectional study conducted at six HIV clinics namely; Muhimbili National Hospital (MNH), Temeke Municipal Hospital, Infectious Diseases Centre (IDC), Mwananyamala Hospital, Mbagala Rangi Tatu Hospital and Sinza Palestina Hospital in Dar es Salaam, Tanzania. The study recruited HIV-positive pediatric patients (aged 3-15 years) attending HIV care and treatment centers (CTC) who were using efavirenz-based cART for at least six months. Pediatric patients with diarrhea, vomiting, those with renal or liver disease were excluded from the study. Pediatric patients using medicines with known potential interactions with efavirenz such as rifampicin, fluconazole and ketoconazole were also excluded from the study.

### Data collection and laboratory analysis

The sample size for this study was calculated based on methods for establishing reference intervals and on previous studies with children of similar age groups which found that 29% and 28% of children had sub and supra-therapeutic EFV plasma concentrations respectively [12, 18, 19]. Taking this into account therefore a sample size of 150 children was proposed with a relative precision of 10% and a confidence interval (CI) of 95%. The children were recruited using consecutive sampling method until a desired sample size was obtained. Interviews were conducted with the aid of structured questionnaires. The interviews extracted data such as demographics, the time efavirenz dose was last taken, data on missed dose(s) in the previous three days, adverse effects and if any over the counter medicine(s) had been taken in the past seven days. A standardized checklist was used to extract more data from patients’ CTC files. Data extracted included weight, WHO clinical stage, current clinical signs and symptoms, cART regimen in use, efavirenz dose in use, previous cART regimen used, last viral load measured and last CD4 cell counts. Clinical examinations were conducted by the attending clinicians, and data on this and prescription information was extracted from the CTC files.

For clinical responses, we were observing; current clinical signs and symptoms, frequency of opportunistic infections, weight, growth/development progress of a child, mid upper arm circumference (MUAC) and WHO clinical staging; weight for age(WAZ) and height for age(HAZ) ≥ − 2 z-score were used to define good clinical outcomes [20, 21]. Weight-for-age and height-for-age were calculated using weight and height for age WHO calculator. Body mass index (BMI) for age percentiles were also calculated using the age-and-sex-specific percentile for BMI using the Centers for Disease Control calculator for children and teens aged 2-19 years [22]. Virologic response was determined using HIV-1 RNA levels (viral load) measurements where a positive response was considered if a child’s viral load was below the cutoff point of 400 copies/mL [10] . With immunological response the focus was on the CD4 cell count/percentage; a CD4 cell count of above the cutoff point of 350 cells/mm^3^ was considered as a positive immunological response as recommended by the NACP guideline for children aged five years and above, and CD4 cell count of 25% or 750cells/mm^3^ for those below five years of age [3].

Blood sampling was done between 12 and 19 h post-dosing (after the interview). This would help to get the relevant information on mid-dosing interval plasma levels because of the long half-life of efavirenz [10]. Venous blood samples were taken for estimation of efavirenz mid-dosing interval plasma concentrations, viral load and CD4 cell count.

Blood samples for viral load and efavirenz plasma concentration analysis was collected in two sterile ethylene diamine tetra-acetic acid tubes and centrifuged within 6 h of sample collection [23]. Centrifugation was at 100% (5100 × 1000 U/minute) for 10 min using Benchtop centrifuge w/6-Well Fixed Angle Rotor model EBA 3S (Hettich Universal, Germany) and later stored at − 80 °C. The viral load measurements were done using Roche Molecular Diagnostic’s COBAS,® TaqMan® Analyzer. The plasma efavirenz levels were analyzed using a validated reverse phase High Performance Liquid Chromatography (HPLC) with ultraviolet detection at Muhimbili University of Health and Allied Sciences (MUHAS) - Swedish International Development Cooperation Agency (Sida) Bioanalytical Laboratory. The individual steady state mid-dosing interval plasma concentration of efavirenz of each child in the study was obtained, the concentrations were checked to see whether they lie within manufacturers’ recommended or published intervals of between 1 and 4 μg/mL [10].

The chromatographic analysis using the HPLC System consisted of auto sampler (SIL-20A, 20 MPa Max Pressure, Shimadzu), UV-detector (SPD-20AV, Shimadzu) and pump (LC-20AT, Shimadzu) with a degasser (DGU-2A3, Shimadzu) and the analytical column was Zorbax Extend C18 (150 × 4.6 mm I.D, 5 μm particle size; Agilent Technologies, Netherlands). Detection wavelength and flow rate were set at 275 nm and 0.8 mL/minute respectively. Carbamazepine was used as the internal standard whereas efavirenz was used as the reference standard. Mobile phase consisted of 25 mM of triethylamine –in-water – acetonitrile mixture (65:35, *v*/v). Method validation of the efavirenz assay was done and both inter-day and intra-day accuracy and precision fulfilled the FDA’s acceptance criteria of being within ±15% for bioanalytical methods [24].

### Statistical analysis

The collected data was entered in SPSS computer statistical package version 21(Copyright 2007, SPSS Inc.; Chicago, IL, USA), followed by data coding, checking and cleaning. Data entry was done twice to ensure appropriate data consistency and quality. Inter-individual pharmacokinetic variability was evaluated using percentage of coefficient of variation (CV %) calculated as a ratio of standard deviation to the mean plasma efavirenz concentration multiplied by 100. Continuous variables were compared using Student’s t-test, while categorical variables were compared using chi-square test. The predicting value of efavirenz plasma concentrations for cART responses (virologic, immunologic and clinical) and CNS adverse effects were determined using univariate and multivariate logistic regression analysis. Variables in univariate analysis with a *p* < 0.2 was included in multivariate analysis to assure that all pertinent and potentially predictive variables are studied. Pearson correlation test was used to analyze the correlation between treatment responses and efavirenz plasma concentration and duration of efavirenz use. A *p*-value of less than 5% *(p* < 0.05) was considered to be statistically significant.

## Results

A total of 327 children were screened from the clinic attendance registers, whereby 151 children were approached at MNH [10], IDC (44), Temeke Hospital [25], Mwananyamala Hospital [26], Mbagala Rangi Tatu Hospital [27] and Sinza Palestina Hospital [5]. Of these 151 children approached, 6 could not be included into the study for various reasons (the parents of five children refused consent, and phlebotomists could not take a blood sample from one). The study thus involved 145 pediatric HIV patients aged between 5 and 15 years with mean ± SD age of 10.83 ± 2.75 years weighing between 13 and 58Kg (mean ± SD weight of 28.30 ± 8.66Kg), with more males (58.6%) compared to females. A total of 43.4% of the children included in the study were orphaned (having lost one parent or both). The most frequent NRTIs used in combination with efavirenz were zidovudine and lamivudine (in 86.2% of children).

At the time of sample collection 44 (30.3%) of the study participants were using cART concomitantly with other drugs (21.4% cotrimoxazole, 2.1% artemether lumefantrine, and 2.8% amoxycillin). None of the patients reported to be taking any traditional medicine (natural health products) and none were on isoniazid preventive therapy (IPT). Using the mid upper arm circumference to evaluate the patients’ nutritional status, 64.6% of the patients had normal nutrition status, whereas 32.6% and 2.8% had moderate and severe malnutrition respectively. Anthropometric weight-for-age Z- scores (WAZ) showed a high proportion of children who were severely undernourished (14.5%) and moderately undernourished (22.1%), with 63.4% having a normal weight. The height-for-age scores showed that 35.1% of the children had varying degrees of stunting (26.2% moderately stunted and 8.9% severely stunted). Using the BMI-for-age percentiles a relatively large proportion of children (31.7%) fell below the 5th percentile indicating underweight among these children. Among the underweight, 54.3% were below the 1st percentile indicating wasting.

The median CD4 T-cell count of the patients was 763 (Interquartile range [IQR] = 498–1069) cells/μL with most of the children (84.8%) having CD4 cell counts of above 350 cells/μL. Virological assessment revealed that 27.7% of patients had detectable viral loads of over 400 copies/mL despite having used cART for a mean ± SD duration of 3.7 ± 2.56 years. All patients had good self-reported adherence to their cART therefore good adherence statuses had been recorded in their CTC-2 cards. Table 1 describes these sociodemographic and clinical characteristics of the children involved in the study.

**Table 1:** Sociodemographic characteristics of patients based on the efavirenz plasma concentrations’ recommended cutoff points

Participant variable		Efavirenz concentration			
		< 1 μg/mL n (%)	1 - 4 μg/mL n (%)	> 4 μg/mL n (%)	*p* value
Sex	Male	11(12.9%)	52(61.2%)	22(25.9%)	0.064
	Females	13(21.7%)	25(41.7%)	22(36.7%)	
Age group (years)	5–10	7(11.1%)	41(65.1%)	15(23.8%)	0.037
	11–15	17(20.7%)	36(43.9%)	29(35.4%)	
Orphan status	Not orphaned	11 (13.4%)	53 (64.6%)	18 (22.0%)	0.006
	Orphaned	13 (20.6%)	24 (38.1%)	26 (41.3%)	
BMI-for-age percentiles	<5th percentile	5 (10.9%)	18 (39.1%)	23 (50.0%)	0.011
	5-85th percentile	19 (19.4%)	58 (59.2%)	21 (21.4%)	
	>85th percentile	0	1 (100%)	0	
Weight for age (WAZ)	Normal weight	15(16.3%)	53(57.6%)	24(26.1%)	0.341
	Moderately undernourished	6(18.8%)	12(37.5%)	14(43.8%)	
	Severely undernourished	3(14.3%)	12(57.1%)	6(28.6%)	
Height for age (HAZ)	Normal height	17(18.1%)	49(52.1%)	28(29.8%)	0.906
	Moderately stunted	5(13.2%)	20(52.6%)	13(34.2%)	
	Severely stunted	2(15.2%)	8(61.5%)	3(23.1%)	
MUAC	Normal nutrition	16(17.2%)	54(58.1%)	23(24.7%)	0.690
	Moderate malnutrition	6(12.8%)	21(44.7%)	20(42.6%)	
	Severe malnutrition	2(50.0%)	2(50.0%)	0(0.0%)	
CD4 cell count (cells/μL)	> 350 cells	17 (13.8%)	68(55.3%)	38(30.9%)	0.108
	< 350 cells	7(31.8%)	9(40.9%)	6(27.3%)	
Viral load (copies/mL)	< 400 copies	7(6.7%)	61(58.1%)	37(35.2%)	0.000
	400–1000 copies	1(33.3%)	1(33.3%)	1(33.3%)	
	> 1000 copies	16(43.2%)	15(40.5%)	6(16.2%)	
Concurrent Medication	No	15 (14.9%)	51 (50.5%)	35 (34.7%)	0.218
	Yes	9 (20.5%)	26 (59.1%)	9 (20.5%)	

*Key: BMI* Body Mass Index, *WAZ* Weight for age Z-scores, *HAZ* Height for age Z-scores, *MUAC* Mid Upper Arm Circumference

### Mid-interval steady state Efavirenz plasma concentration

The median time for sample collection for mid-interval efavirenz plasma concentration was 15.6 [IQR = 14.5–17.2] hours. The overall median mid-interval steady state efavirenz plasma concentration was found to be 2.56 [IQR = 1.5–4.6] μg/mL for all the patients. There was no significant difference in efavirenz plasma concentrations for patients whose sampling times were 12 ± 2 and17 ± 2 h post-dosing (median 2.69 [IQR = 21.7–4.4] versus 2.39 [IQR = 1.4–5.1] hours respectively). Results based on the mean ± SD plasma efavirenz concentration of 4.41 ± 4.89 μg/mL, the calculated coefficient of variation was found to be 111% for inter-patient variability of efavirenz plasma concentration.

Only 53.1% of the children were within the recommended efavirenz plasma concentration levels of 1–4 μg/mL with 16.6% having concentrations below 1 μg/mL. Table 1 summarizes the sociodemographic characteristics of the patients based on the recommended plasma efavirenz concentrations cutoff points of < 1 μg/mL, 1–4 μg/mL and > 4 μg/mL. Majority of male participants (61.2%) had efavirenz concentrations between 1 and 4 μg/mL compared to female participants, however this was not statistically significant (*X*^2^ (*N* = 145) = 5.48, *p* = 0.064). The 5–10 years age group had a significantly larger number of patients (65.1%) with plasma efavirenz concentrations within the 1–4 μg/mL interval compared to those aged 11–15 (43.9%), whereas those aged 11–15 had significantly more children with efavirenz concentrations below 1 μg/ml compared with children aged 5–10 years (20.7% versus 11.1%) (*X*^2^ (N = 145) = 6.57, *p* = 0.037). It was found that 55.3% of the participants with CD4 T-cell counts greater than the cutoff point of 350 cells/μL (good immunological response) had efavirenz plasma concentrations between 1 and 4 μg/mL, whereas 30.9% of the patients with CD4 below 350 cells/μL had plasma efavirenz levels < 1 μg/mL. Efavirenz plasma concentrations of > 1 μg/mL was found to be associated with good immunological response (OR = 3.1 {95% CI: 0.86–8.95}, *p* = 0.051).

**Fig. 1:**
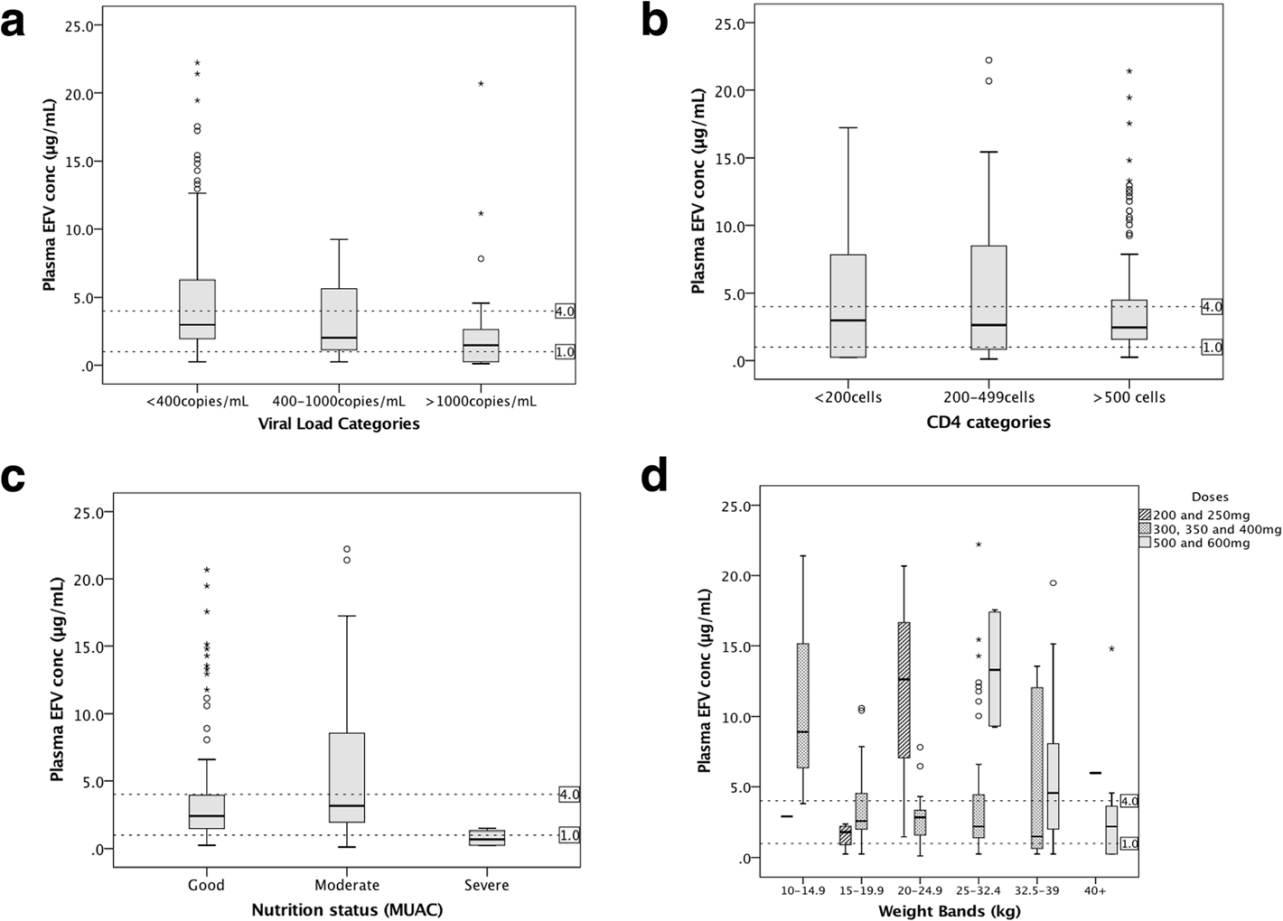
Box plots showing association between efavirenz plasma concentrations and the virological, immunological and clinical outcomes. Key: These graphs show efavirenz mid-dosing concentrations in children; central line represents median values while box and whiskers represent interquartile range and 10th –90th percentile, respectively, and individual points are outliers. Dotted lines represent 1 μg/mL and 4 μg/mL (Therapeutic range). **a** Efavirenz concentrations vs Viral load categories (< 400 copies/mL, 400–1000 copies/mL and > 1000 copies/mL). **b** Efavirenz concentrations vs CD4 categories (< 200 cells/μL, 200–499 cells/μL, > 500 cells/μL). **c** Efavirenz concentrations vs Nutritional status (MUAC). **d** Efavirenz concentrations vs weight bands in Kg and efavirenz doses in mg

The study found that a statistically significant association (*p* < 0.001) exists between efavirenz plasma concentrations and viral load. Of the 105 participants with good virologic response (viral load < 400 copies/mL) 58.1% had therapeutic efavirenz plasma concentrations (1–4 μg/mL) while 35.2% were found to have efavirenz plasma concentrations above 4 μg/mL. Efavirenz plasma concentration of > 1 μg/mL was therefore found to be associated with virologic success (OR = 9.5{95% CI: 3.6–25.1}, *p* < 0.01). Virologic failure was found to be 70% among those with sub-therapeutic levels compared to only 20.5% and 15.6% in the therapeutic and supra-therapeutic levels. Figure 1 shows the relation between efavirenz concentrations and the virological, immunological and clinical outcomes.

Univariate and multivariate analysis was done with the inclusion of relevant factors in predicting outcomes associated with efavirenz levels below the recommended plasma concentrations of < 1 μg/mL (Table 2). It was found that CD4 cell counts, viral load levels, sex and child age group were some of the risk factors associated with very low plasma efavirenz concentrations (*p* < 0.2) with 1 degree of freedom (df). In the multivariate analysis, only the viral load maintained statistical significance with a *p*-value of < 0.001 showing that those with low efavirenz plasma concentrations (< 1 μg/mL) are 11 times more likely to have detectable viral loads of more than 400 copies/mL.

**Table 2:** Univariable and multivariable logistic regression analysis to assess the risk factors associated with efavirenz plasma concentration below 1 μg//mL among children being treated with efavirenz based cART

Variable	Number of efavirenz samples N	Efavirenz < 1 μg//mL N (%)	Univariate analysis		Multivariate analysis	
			Crude OR (95% CI)	p-value	Adjusted OR (95% CI)	*p*- value
Sex						
Male	85	11 (12.9)	1		1	
Female	60	13 (21.7)	1.86 (0.77–4.49)	0.168	1.83 (0.68–4.95)	0.232
Age group (years)						
5–10	63	7 (11.1)	1		1	
11–15	82	17 (20.7)	2.09 (0.81–5.41)	0.128	2.07 (0.69–6.15)	0.192
Orphan status						
No	82	11 (13.4)	1			
Yes	63	13 (20.6)	1.67 (0.69–4.05)	0.249		
CD4 (cells/μL)						
> 350	123	17 (13.8)	1		1	
< 350	22	7 (31.8)	2.91 (1.03–8.17)	0.043	0.79 (0.22–2.84)	0.718
Viral Load (copies/mL)						
< 400	105	7 (6.7)	1		1	
> 400	40	17(42.5)	10.3 (3.84–27.86)	0.000	11.0 (3.66–33.09)	0.000
BMI-for-Age Percentiles						
5th – 85th	99	19 (19.2)	1			
< 5th	46	5 (10.9)	0.51 (0.18–1.47)	0.215		
Height for age						
Normal height	94	17 (18.1)	1			
Moderately stunted	38	5 (13.2)	0.68 (0.23–2.01)	0.493		
Severely stunted	13	2 (15.4)	0.82 (0.17–4.06)	0.811		
Weight for age						
Normal weight	92	15 (16.3)	1			
Moderate malnutrition	32	6 (18.8)	1.18 (0.42–3.37)	0.751		
Severe malnutrition	21	3 (14.3)	0.86 (0.22–3.27)	0.820		

*Key: cART* combination antiretroviral therapy, *OR* Odds Ratio, *CI* Confidence interval, *BMI* Body mass index

Majority of study participants (86.2%) had not experienced known efavirenz associated adverse drug reactions(ADRs) (CNS and/or skin rash) within the past six months prior to the study. Majority of patients (65.5%) who reported CNS ADRs had efavirenz plasma concentration between 1 and 4 μg/mL. Over 54% of those who reported skin rash within the past six months were found to have efavirenz concentrations within the recommended therapeutic range of 1–4 μg/mL (*X*^2^ (*N* = 145) = 2.61, *p* = 0.856).

## Discussion

This study has shown that the overall median for all the participants’ efavirenz plasma concentration to be within the recommended therapeutic range, however, there was very high inter-individual variability (111%). The high variability resulted in 16.6% of the patients having sub-therapeutic efavirenz plasma concentrations and 30% with supra therapeutic and potentially harmful plasma concentrations. Virologic failure was very high (70%) among those with sub-therapeutic levels compared to those with therapeutic and supra-therapeutic levels. The probability of having virological failure among those with sub-therapeutic levels was 11 times more compared to those with therapeutic and supra-therapeutic levels.

The median efavirenz levels from this study are comparable to the findings reported in another study whereby a median of 2.8 μg/mL was observed with the samples having been collected 8–20 h post dosing [26]. The proportion of patients found with recommended adequate efavirenz plasma concentrations in this study was 53.1%. This proportion is much lower than that seen in other studies where the proportions of patients within the recommended therapeutic levels ranged from 60 to 71% [12, 27, 28]. However, a meta-analysis by Bouazza et al. showed that the probability of being within the recommended therapeutic range varied between 56 and 60% regardless of the fixed dose combination [29]. These results are more in keeping with the findings from our study.

We observed a wide range of inter-individual variability of 111% in the plasma concentrations placing a very large proportion of the children outside the therapeutic range of 1–4 μg/mL. Such wide inter-individual variabilities have also been seen by other studies [10, 28, 30]. The variability may be attributed to the growth and development processes which are still ongoing among pediatric patients impacting the maturity of metabolic organs such as liver and kidneys, feeding patterns affecting drug absorption and hence bioavailability, maturation of hepatic enzymes and variation in drug elimination [31, 32]. Variability can furthermore be attributed to genetics, particularly single nucleotide genetic polymorphism of the gene for *CYP2B6* enzyme responsible for efavirenz metabolism [33]. Higher mean plasma efavirenz concentrations in Tanzanians were observed compared to Ethiopians, suggesting slow efavirenz metabolism among Tanzanians [34]. This is consistent with our findings in which 30.3% of our study participants were found to have efavirenz plasma concentrations above the recommended therapeutic interval even though these patients had been on efavirenz doses prescribed in accordance with the recommended weight bands. Therefore, based on our findings we believe that some of our study participants might be slow efavirenz metabolizers due to genetic polymorphism leading to higher than recommended plasma concentrations.

Our study found that a significantly strong association exists between efavirenz plasma concentration and virologic response. Out of the 24 participants (16.6%) with sub-therapeutic plasma efavirenz concentrations 17 (70.8%) were found to have poor virologic treatment response compared to only 20.5% and 15.6% of those with therapeutic and supra-therapeutic efavirenz plasma concentrations respectively. Patients with plasma efavirenz concentrations of below 1 μg/mL are 11 times more likely to have detectable viral load levels. Our findings are consistent with findings in other studies which also found the existence of significant association between efavirenz plasma concentrations and virologic treatment response [15, 35–39]. Various studies have also reported sub-therapeutic concentrations of varying prevalence, relating it to non-adherence to treatment and associating it with poor treatment response [28, 40].

Poor virologic treatment response was observed in 15.6% of participants with supra-therapeutic efavirenz concentrations. Viral resistance may be the reason for these patients to have poor virologic response despite the fact that they were found to have supra-therapeutic concentrations. The high proportion of children with supra-therapeutic efavirenz concentrations suggests that the doses of efavirenz given to our pediatric patients may not be optimal in providing the necessary concentrations for therapeutic needs putting them at a higher risk for CNS toxicity. Supporting our findings with the findings by Mukonzo et al., we believe that our pediatric patients are being given efavirenz at doses larger than their therapeutic needs exposing them to more potential risk of CNS adverse drug reactions (40). These findings also emphasize the importance of monitoring efavirenz plasma concentrations to ensure that pediatric patients whose pharmacokinetics are subject to constant changes due to growth and development benefit maximally from cART.

Clinical response based on weight for age, height for age and MUAC revealed a non-significant association between efavirenz plasma concentration and clinical treatment response. The study by Mutwa et al. conducted in Rwandan children reported that a poor clinical response based on WAZ or HAZ is associated with a poor immunological recovery and virological failure [41].

It is known that children in sub-Saharan Africa have high levels of malnutrition with low weight-and-height for age indicating large proportions of wasting and stunting. Studies within Tanzania and neighboring countries have reported high proportions of poor nutritional status among HIV children [41–43]. Similar proportions have been seen in this study where a large number of children were either stunted or underweight, and among the underweight a significant proportion were wasted. The poor nutritional status of the children could potentially have an impact on the immunological and pharmacological responses to cART.

## Conclusion

Our study has demonstrated a wide inter-individual variability in efavirenz plasma concentrations among Tanzanian pediatric patients in routine clinical practice with a little over half of the children within the recommended therapeutic range. Virologic failure is very high in children with sub-therapeutic levels with the probability of having virological failure among those with sub-therapeutic levels being 11 times more compared to those with therapeutic and supra-therapeutic levels. A large proportion of children have poor nutritional status. Children with concentrations outside the therapeutic window pose a risk of treatment failure due to sub-therapeutic plasma concentrations and risk for CNS adverse drug reactions resulting from supra-therapeutic levels. This emphasizes the importance of conducting therapeutic drug monitoring to ensure better treatment success particularly for pediatric HIV patients.

## Abbreviations

AIDS: Acquired Immunodeficiency Syndrome
BMI: Body mass index
cART: Combination antiretroviral therapy
CNS: Central Nervous System
CTCs: Care and Treatment Centers
CV: Coefficient of Variation
HAZ: Height for age
HIV: Human immunodeficiency virus
HPLC: High-Performance Liquid Chromatography
ICD: Infectious Disease Center
IRB: Institutional Review Board
MNH: Muhimbili National Hospital
MUAC: Mid upper arm circumference
MUHAS: Muhimbili University of Health and Allied Sciences
NACP: National AIDS control Program
NNRTI: Non-Nucleoside Reverse Transcriptase Inhibitor
NRTI: Nucleoside Reverse Transcriptase Inhibitors
WAZ: Weight for Age
WHO: World health organization
